# Automated drosophila heartbeat counting based on image segmentation technique on optical coherence tomography

**DOI:** 10.1038/s41598-019-41720-1

**Published:** 2019-04-03

**Authors:** Chia-Yen Lee, Hao-Jen Wang, Jheng-Da Jhang, I-Chun Cho

**Affiliations:** 1 Department of Electrical Engineering, National United University, Miaoli, ROC Taiwan; 2 Department of Biomedical Engineering, National Taiwan University, Taipei, ROC Taiwan; 3 Medical Physics and Informatics Laboratory of Electronics Engineering, National Kaohsiung University of Science and Technology, Kaohsiung, ROC Taiwan

## Abstract

Drosophila and human cardiac genes are very similar. Biological parametric studies on drosophila cardiac have improved our understanding of human cardiovascular disease. Drosophila cardiac consist of five circular chambers: a conical chamber (CC) and four ostia sections (O1–O4). Due to noise and grayscale discontinuity on optical coherence tomography (OCT) images, previous researches used manual counting or M-mode to analyze heartbeats, which are inefficient and time-consuming. An automated drosophila heartbeat counting algorithm based on the chamber segmentation is developed for OCT in this study. This algorithm has two parts: automated chamber segmentation and heartbeat counting. In addition, this study proposes a principal components analysis (PCA)-based supervised learning method for training the chamber contours to make chamber segmentation more accurate. The mean distances between the conical, second and third chambers attained by the proposed algorithm and the corresponding manually delineated boundaries defined by two experts were 1.26 ± 0.25, 1.47 ± 1.25 and 0.84 ± 0.60 (pixels), respectively. The area overlap similarities were 0.83 ± 0.09, 0.75 ± 0.11 and 0.74 ± 0.12 (pixels), respectively. The average calculated heart rates of two-week and six-week drosophila were about 4.77 beats/s and 4.73 beats/s, respectively, which was consistent with the results of manual counting.

## Introduction

Optical coherence tomography (OCT) systems have an advantage of high-resolution imaging. Its resolution ranges from a few micrometres to a few hundred micrometres, 10 to 100 times better than that of ultrasound, computed tomography and magnetic resonance imaging (MRI)^1^. The OCT system has a high scanning rate, and its signal is attenuated with depth due to scattering in the tissue. Therefore, image depth resolution is typically limited to 2–3 mm. Such non-invasive imaging technique has been used in many clinical studies such as ophthalmology, cardiovascular disease and dermatology^2,3^.

Drosophila can be used as an important model for investigating heart diseases because there are about 75% of human disease-causing genes have functional homolog in Drosophila. In addition, they have a large number of offspring and the number of individuals with genetic variation is relatively large. There are some methods for obtaining the heart structure of drosophila and observing its heartbeat include histology^4^, microscopy^5,6^, electrical pacing stress^7^ and multi-electrode array system^8^.

Histology may be used to obtain high-resolution information of drosophila cardiac structure, but this method may destroy the cardiac structure and is unable to obtain heartbeat data from live drosophila. Microscopy may be used to observe chamber structure and beating behaviour of drosophila cardiac; however, the drosophila shell has a highly scattering texture, which results in light not easily penetrating the drosophila for *in vivo* imaging, and it is necessary to remove the drosophila shell to observe the cardiac structure.

Since 2006, OCT technology has been used in drosophila research. For example, a research team led by Prof. J. A. Izatt used OCT to observe the heartbeats of drosophila conical chambers^9,10^. In 2009, the research team of Michelson Diagnostics Inc. developed an OCT system for imaging of drosophila cardiac conical chambers^11^. The research team of Prof. J. G. Fujimoto used an OCT system to study Alzheimer’s disease in drosophila genetic research^12^. Some researchers also employed an OCT system to calculate drosophila cardiac wall velocity and used nanoparticles as an image contrast enhancer to measure body fluid velocity in drosophila cardiac^13^; Similar to an ultrasound detection system, an OCT system can provide B-mode images, and its axial resolution is up to 1–2 μm. Because of the high transmittance and obtainable cross-section image characteristics of drosophila, OCT is more suitable than other imaging modalities for use in drosophila. Although OCT technology is able to reconstruct tissue structure images of 2–3 mm, cardiac curvature, system sensitivity and resolution may decrease as image depth is increased. Therefore, current use of OCT for drosophila study is focused on dynamic observation of CC, but most pathogenic genes may affect not only the beating behaviour of CC but also the beating behaviour of other four ostia (O1, O2, O3 and O4).

M-mode imaging is mostly used when OCT is used to observe drosophila cardiac^10,14^, which is suitable for observing changes in time-series images without the trouble of time-consuming manual counting. However, M-mode images need to manually select the position to calculate the heart rate. This assignment depends on the operator’s experience, and different operators may result in different heart rate calculations. Furthermore, the noise in the image will also make the calculation inaccurate.

Thus, image segmentation techniques may be used to segment the drosophila chamber to achieve better results without the need of transforming two-dimensional images into one-dimensional ones. The threshold method for image segmentation is suitable for separating foreground and background; this method is extremely useful for rough segmentation, but not suitable for locally complicated images. The watershed^15^ is a well-known segmentation method; this method is capable of fine local segmentation, but still has problems of over segmentation and block merging, especially for OCT images with chromatic dispersion and high noise. In addition, the internal structure of drosophila images is complex, and there are many bright and dark blocks, which are difficult to distinguish effectively. There are many methods used for clustering. For example, Manjunath and Chellappa^16^ combined texture and a Markov field model to complete clustering. Some researchers proposed a ‘cut’ method to separate foreground and background. Wu and Leahy^17^ tried to cut the best results by means of energy minimization. Shi and Malik^18^ proposed the well-known energy minimization optimum solution (normalize cut). Comaniciu^19^ proposed a mean shift algorithm based on clustering. On the other hand, k-means is a widely used iteration-based clustering method^20,21^. In later studies, researchers applied the k-means clustering method to local solutions and produced super-pixel^22^. However, most clustering methods rely on colour classification of natural images, and using clustering alone is not enough to achieve good results in biomedical imaging. In addition, although there are many segmentation algorithms proposed in OCT ophthalmologic imaging, but not for the drosophila cardiac, most of them are for the retina, and the segmentation technique is different between the retina and the drosophila cardiac.

Bagci^23^ used the correlation of axial A-scans to extract the boundaries between each pair of adjacent bright and dark regions of six retinal layers in OCT image and the same method is used in many studies for retinal thickness analysis in OCT datasets^23,24^. Ghorbel^25^ proposed an automated method that based on global segmentation algorithms for segmentation of eight retinal layers in Heidelberg spectralis SDOCT images. In addition, the Kalman filter was designed to simulate the approximate parallelism between photoreceptor segments and to detect them.Fang *et al*.^26^ proposed a segmentation based sparse reconstruction (SSR) model for retinal OCT image reconstruction, using segmentation layer information to improve the effectiveness and efficiency of the reconstruction model. This study effectively preserves the anatomical and pathological structures of retinal in OCT images using the SSR method.

The segmentation topic that is more relevant to our topic is the retinal cyst segmentation of OCT images, Oguz *et al*.^27^ a knowledge-based approach that uses cost functions and graph-based segmentation techniques to provide a solution to this problem. The study validated the results on two publicly available datasets, and the volume similarity error was significantly reduced from 81.3 ± 56.4% to 22.2 ± 21.3% compared to the previous method (paired t-test, p ≪ 0.001). Abdolreza Rashno *et al*.^28^ proposed a fully automated algorithm for segmenting cystic areas in OCT retinal images from patients with diabetic macular edema. The OCT image is segmented using a novel neutrosophic transformation and a graph-based shortest path method. The proposed algorithm for the Duke, Optima, and University of Minnesota (UMN) datasets, the proposed algorithms also achieved sensitivity of 67.3%, 88.8%, and 76.7%, respectively.

In 1988, Sethian and Osher proposed a level set algorithm^29^, which has been widely promoted and used in geometric calculation, hydrodynamics, image processing and computer vision. Caselles^30^ and Kichenassamy^31^ *et al*. added a snake model into the level set algorithm. Chan and Vess^32^ modified the previous variable model that depended on gradient and that easily converged on a local solution, and proposed a region-based two-phase level set algorithm, but there is still a problem in re-initialisation. The method proposed by Li^33^ avoids the need to periodically re-initialise the distance function, but instead adds restrictions in each evolution so that the slope of the function is not too large. In the graph theory, it only needs to select seed points for achieving semi-automatic calculation of heart chamber parameters^34^. Unlike many studies, it is only visually counting the heart rate of the conical chamber^35^. We employed sagittal plane images for the measurement of the conical chamber and four ostia portions parameters. We would like to achieve automatic chamber segmentation to process each chamber at the same time and evaluate the quality of segmentation results in detail.

## Materials

OCT images were used to observe drosophila cardiac characteristic of different ages. Wild-type Drosophila were raised at room temperature (25 °C) until maturation. The ages of the drosophila included: 2 weeks, 4 weeks and 6 weeks. Epoxy resin was used to cause drosophila be unconscious so as to secure them onto a slide. The use of epoxy resin did not affect the heartbeat performance of the drosophila. On the slide, the drosophila should be straight with the cardiac facing up, so that the OCT system can accurately capture a whole cardiac image of the drosophila (some non-completely unconscious drosophila may tremble during the OCT scan).

**Figure 1:**
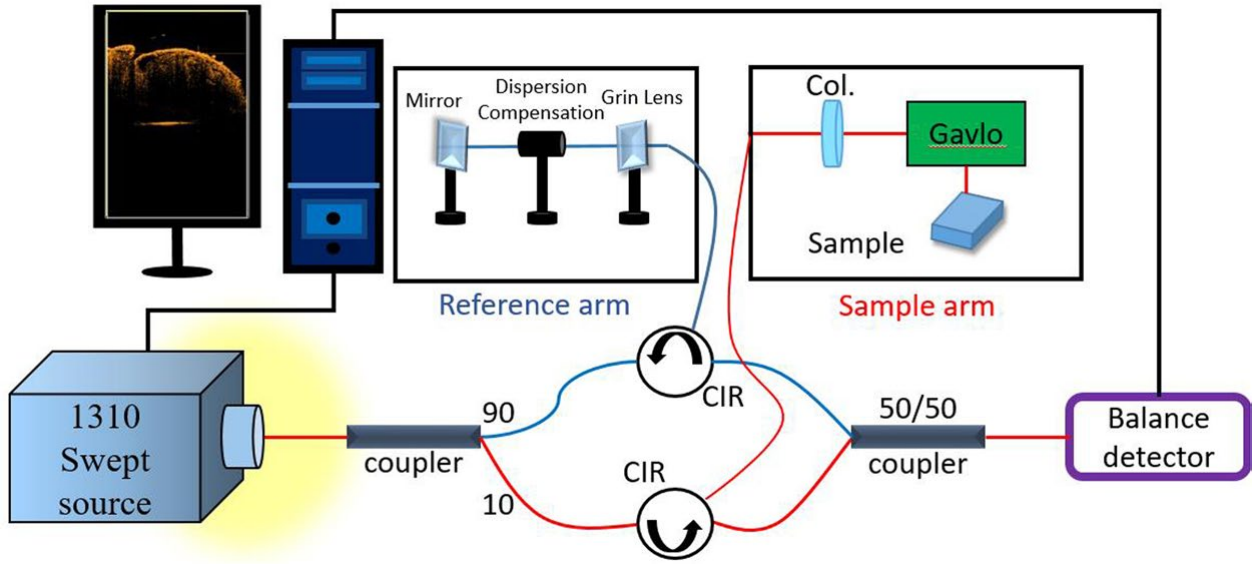
SS-OCT system structure. (1) CIR: optical circulator; (2) Col.: optical collimator; (3) dispersion Compensation: dispersion compensating mirror.

In this paper, an SS-OCT system was used for drosophila cardiac imaging. The system is shown in Fig. 1. SS-OCT system includes a swept-frequency laser structure (centre wavelength: 1.3 μm; scanning frequency: 100 kHz; FWHM: 110 nm), connected to a light source; the system simultaneously comprises a 90/10 and 50/50 optical fibre coupler and two optical circulators. On the reference end, a mirror and a dispersion compensator was used; the mirror were used to reflect light on the reference end, whereas the dispersion compensator was used to compensate for chromatic dispersion caused by a lens on the sample end. On the sample end, light was directed through a two-dimensional scanner and a focusing lens to perform transverse scanning, while reflected light on the reference end and backscattered light on the sample side were recombined by the 50/50 optical coupler to form an interference signal. The interference signal is finally received by a balanced detector and converted by an A/D converter and subsequent digital processing. Each two-dimensional image (B-mode) is configured to contain 1000 A-mode segments. The imaging speed of the OCT system is 100 frames/s. Using high-frequency imaging, the system can obtain two-dimensional sequential images of drosophila sections without distortion.

## Results and Discussion

**Figure 2:**
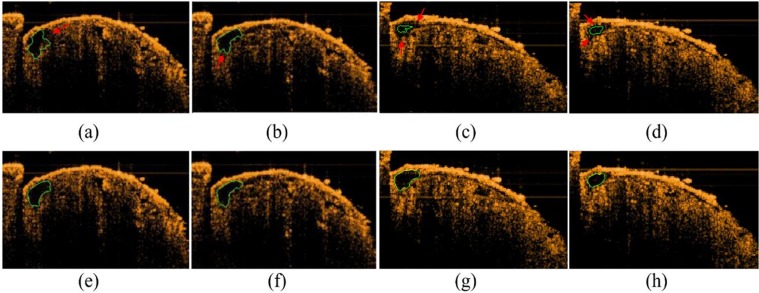
Shape Prior-based Level Set Model; (**a**–**d**): results of the Chan-Vese level set method; (**e**–**h**): results of the proposed level set method.

The method proposed in this study improved the situation when the chamber boundary was unclear, weak boundary, as indicated by the red arrow in Fig. 2. Giving a shape prior, the segmented chamber results were closer to the ground truth. As shown by the green boundary in Fig. 2, in which a state of a weak boundary is exhibited on the right side of the main chamber. Figure 2(a–d) showed four drosophila cardiac segmentation results derived by the Chan-Vese level set method, and Fig. 2(e–h) gave the corresponding segmentation results by the proposed algorithm. The Chan-Vese level set method were not detect the weak boundary.

Figure 2(e) shows a significant improvement in the right side of the chamber, indicating that the contours obtained by the proposed method can converge at the real edge. When a new energy term was added into the algorithm but the convergence condition remains unchanged, the restriction of energy convergence would become more stringent, thereby making evolution closer to the edge. Conversely, if strict convergence conditions were set in the Chan-Vese level set method, the evolution of that would be very likely to exceed the real edge in case of weak edge. The contour evolution in Fig. 2(b) was quite close to the real edge, but the contour was not smooth because of many corner angles. This is because the actual chamber of drosophila is tubular, the Chan-Vese level set method was inconsistent with the actual shape. After shape restriction, excessive corner angles were removed, thereby obtaining a smooth contour shown in Fig. 2(f).

Figure 2(c,d) is another drosophila. It can be seen that the segmentation result using the Chan-Vese level set method cannot be close to the real boundary due to the influence of noise and weak boundary. The method proposed in this study can obtain better chamber segmentation results, as shown in Fig. 2(c,d).

**Figure 3:**
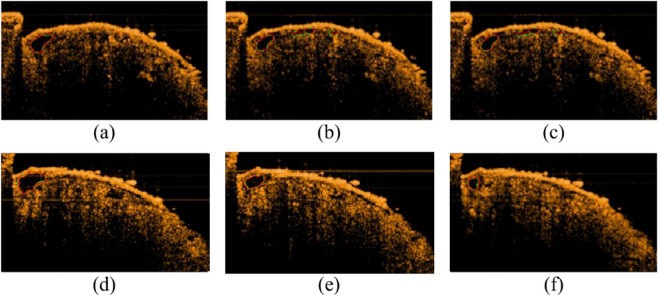
Imaging results after segmentation: red line representing the ground truth, green line representing the propsoed algorithm results.

In this study, the age of drosophila was generally 2 weeks or 6 weeks. In addition to cardiac parameters, imaging results were evaluated to investigate the difference between segmentation results and the actual situation, as shown in Fig. 3. The ground truth was delineated by researchers who had at least 2 years of experience in drosophila imaging to verify the correctness of the results of the proposed algorithm.

### Performance analysis

Dice coefficient and contour mean distance were used to evaluate the difference between the ground truth contours and the results. The Dice coefficient in eq. (1) can be viewed as a similarity measure of sets with high sensitivity. The coefficient is used as an area coverage rate of two contours in the evaluation.

1 $${\rm{Dice}}\,{\rm{coefficient}}=\frac{2|A\cap B|}{|A|+|B|}$$

where |*A*| and |*B*| are the cardinalities of the two sets. ∩ represents the intersection.

Another evaluation method was based on the difference of contours. For example, the shortest distance from each point on the contour to the standard answer can be calculated through eq. (2), thereby obtaining the average distance between each point and the standard answer.

2 $$\frac{1}{|S(A)+S(B)|}(\sum _{{s}_{A}{\epsilon }S(A)}d(s,S(B))+\sum _{{s}_{B}{\epsilon }S(B)}d({s}_{B},S(A)))$$

where |*A*| and |*B*| are the cardinalities of the two sets. *s*_*A*_ represents the contour or surface of A and *s*_*B*_ represents the contour or surface of B $|S(A)+S(B)|$ represents the total number of pixels in the contour or surface of A and B.

To evaluate the performance of the proposed approach on OCT drosophila images, there are two assessment. In the first assessment, the similarity coefficient (Dice) and distance difference indicate whether the results is good. The performance of segmentation results obtained by the proposed method are shown in Table 1. Clearly, the proposed can be applied to the different chambers.

**Table 1:** Evaluation of similarity between manually delineated chamber and results of the proposed algorithm.

	Similarity coefficient (Dice)	Mean distance (Pixel)
Conical chamber	0.83 ± 0.09	1.29 ± 0.59
Second chamber	0.75 ± 0.11	1.47 ± 1.25
Third chamber	0.74 ± 0.12	0.84 ± 0.60

**Figure 4:**
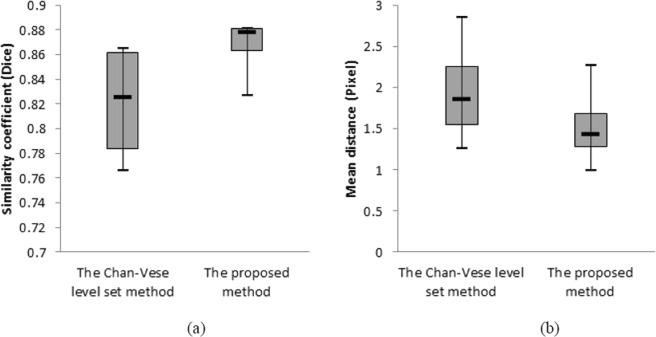
(**a**) The boxplots for the similarity coefficients between the corresponding mean manually delineated boundaries and the segmented results. (**b**) The boxplots for the mean distances between the corresponding mean manually delineated boundaries and the segmented results.

The second assessment is to further justify which segmentation results of the proposed algorithm and the Chan-Vese level set method is better. Figure 4 shows that the boxplots for the mean distances between the corresponding mean manually delineated boundaries and the segmented results by the Chan-Vese level set method and the proposed method. It is evident that the proposed method is better. The main different in the segmented contour is that the proposed method can overcome the problem of the weak boundaries.

### Heart rate counting

The heart rate of normal drosophila is about 4~6 beats/s. In this study, the change in contour area was converted to a one-dimensional signal, and the peak was automatically detected as the heart rate of the drosophila. The average calculated heart rates of two-week and six-week drosophila were about 4.77 beats/s and 4.73 beats/s, respectively, which was consistent with the results of manual counting, i.e., the heartbeat rate of Drosophila is approximately 4~6 beats/s. The two-week drosophila is younger and has a faster heart rate than the six-week drosophila.

## Conclusion

Previous OCT studies on drosophila parameters were usually dependent on manual setting, which was inefficient and time consuming. In the present study, we used the OCT system to obtain drosophila images and developed an algorithm to automatically segment drosophila chambers and calculate the heart rate of drosophila, thus achieving higher efficiency and accuracy than manual counting. First, image was pre-processed and substituted into a level set algorithm to automatically segment the chamber, so as to move the contour closer to the real edge. Further modify the level set algorithm equations at different time points, combine training results with principal component analysis as a priori shape^36^, Building Shape Prior-based Level Set Model to improve the accuracy of the results. The proposed image segmentation algorithm can obtain the drosophila ventricular boundary near the true edge, which makes the automatic calculation of drosophila heartbeat rate more accurate.

## Methods

This study proposes a novel segmentation algorithm, which based on level set algorithm can accurately segment the chamber of drosophila. The process is divided into two parts including drosophila chamber segmentation combined with morphological method, drosophila chamber detection, and using PCA algorithm to adjust the contour for optimal chamber segmentation. The segmentation results can be further applied to the automated counting of drosophila heartbeats.

### Automatic chamber segmentation

To calculate drosophila cardiac parameters, image processing technology was used for automatic segmentation of drosophila cardiac chambers. First, chambers were located automatically to obtain an initial contour. Subsequently, the level set algorithm was used to perform contour correction.

#### Drosophila chamber delectation

**Figure 5:**
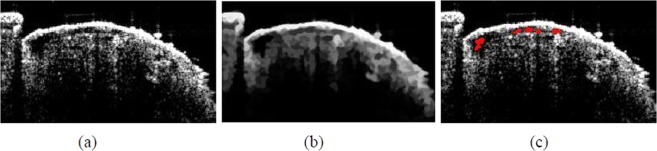
Back-based chamber detection (**a**).

According to the OCT image characteristics of drosophila, blood flow echoes in drosophila chamber are small and being low in the grayscale value. Chamber detection is based on the local minimum method. However, if the local minimum is taken directly, it would be too sensitive and susceptible to noise. Therefore, the opening and closing of a morphology operator were used for reconstruction before processing. The opening operator causes peaks to be smooth in the images, while the closing operator causes peak-to-valley portions to be smooth. Using the local minimum after peak smoothing may increase the robustness of the method and reduce noise interference. On the basis of on the local minimum and the distance relationship between the chamber and the back, we can screen out local minimums representing different chambers, as shown in Fig. 5.

#### Drosophila chamber segmentation-Shape Prior-based Level Set Model

The boundary of local minimum area in the chamber was used as an initial contour, which provides to level set algorithm^37^ for chamber segmentation. The method would segment the contour smoothly to be close to the real boundary by minimising the energy function as similar as gradient flow derivation. The time variable *t* ∈ [0, ∞) was used to solve the eq. (3):

3 $$\frac{\partial \varnothing }{\partial {\rm{t}}}=-\frac{\partial \varepsilon }{\partial \varnothing }$$

where ∅ is the level set function, and $\varepsilon$ is the energy of function.

Given an initial function ∅_0_, a gradient descent method was used for iterative calculation of this time-dependent function. The energy function is expressed as eq. (4):

4 $${\rm{\varepsilon }}(\varnothing )={\rm{\mu }}{R}_{p}(\varnothing )+\lambda {L}_{g}(\varnothing )+\alpha {A}_{g}(\varnothing )$$

where μ, *λ* > 0, $\alpha {\epsilon } {\mathcal R}$ are the coefficients of the energy function, *R*_*p*_ is the level set regularization term.

5 $${R}_{p}(\varnothing )\triangleq {\int }_{{\rm{\Omega }}}P(|\nabla \varnothing |)dx$$

6 $$p(s)=\{\begin{array}{cc}\frac{1}{{(2\pi )}^{2}}(1-\,\cos \,2\pi s) & {\rm{if}}\,s\le 1\\ \frac{1}{2}{(s-1)}^{2} & {\rm{if}}\,s\ge 1\end{array}$$

Equation (6) shows that the smaller the *p* value is when s approaches 1 or 0, that is, when the slope of the zero level function is equal to 1, which conforms to the characteristics of the distance function, there is no limit energy. On the other hand, when the slope is 0, it is to avoid unnecessary energy for the flat area away from the zero level. ${L}_{g}$ represents the boundary term, the line integral of the zero level, and its purpose is that ${L}_{g}$ has a minimum energy when the zero level contour of $\varnothing$ is located at the object boundaries, as shown in eq. (7):

7 $${L}_{g}(\varnothing )\triangleq {\int }_{{\rm{\Omega }}}g{\rm{\delta }}(\varnothing )|\nabla \varnothing |dx$$

where *δ* is the Dirac delta function that the energy *L*_*g*_ (∅) computes the line integral of the function *g* along the zero level contour of ∅. Ω is a contour C:[0, 1] by parameterizing the zero level set. *g* is defined as an edge indicator function:

8 $$g\triangleq \frac{1}{1+|\nabla {G}_{\sigma }\,\ast \,I|}$$

where *I* is the image, *G*_*σ*_ is a Gaussian kernel with a standard deviation *σ*.

If the initial contour is in the flat region, the function will not change in energy, and *A*_*g*_ (∅) is an additional force that moves the initial zero level to the desired position. As shown in eq. (9):

9 $${A}_{g}(\varnothing )\triangleq {\int }_{{\rm{\Omega }}}(-\,\varnothing )dx$$

where $H$ is the Heaviside function. The role of $g$ in this energy term ${A}_{g}$ is that when the zero level contour arrives at object boundaries where g takes smaller values to slow down the shrinking or expanding of it.

If the target contour has a weak edge, the level set algorithm cannot achieve effective convergence at the target boundary. In this paper, we utilize principal component analysis to train a template model^38^ and combined the level set algorithm to achieve effective convergence at the edge.

**Figure 6:**
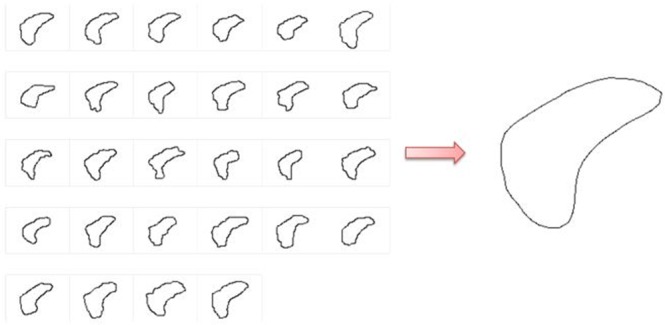
Model establishment by PCA.

Using supervised learning, when the main chamber of the drosophila cardiac expands to a large area, the lower half of the main chamber contour has poor resolution, weak boundary, or even missing boundary. In order to process the contour at a specific-time, correct the original level-set cost function error, via add a trained model as a priori model. Establish the specific shapes of the conical chamber and reference the corresponding manually delineated boundaries defined by two experts. Each contour was defined as a dimension. PCA was used to reduce dimensions and to retain the component which has the largest feature representative. Figure 6 shows the 28 corresponding manually delineated boundaries as input training samples to obtain a training contour.

After using PCA to reduce dimension, the points of each contour must keep same and correspond to each other. Hence, instead of using the entire contour, express the complete contour by sample point and set the amount of sample points of each data in the same, as eq. (10):

10 $${X}_{i}={({x}_{i1},{y}_{i1},{x}_{i2},{y}_{i2},\cdot \cdot \cdot ,{x}_{in},{y}_{in})}^{T}$$

where *x* and *y* represent positions on the horizontal axis and vertical axis; n represents the n^th^ sampling point in the contour.

In practice, 45 points were sampled for each contour according to conical chamber size. The sampled points can reflect the shape of the chamber and avoid dense information. However, the conical chamber in different images may not in the same position. The centre of the contour should be calculated first, and set the difference between each sampling point and contour centre point as the coordinates:

11 $$\widehat{{X}_{i}}={X}_{i}-\bar{X}$$

Calculate a feature vector by coordinates:

12 $${\rm{S}}{p}_{k}={\lambda }_{k}\,{p}_{k}$$

where S represents a covariance matrix of $\hat{{\rm{X}}}$; another condition in eq. (12) is ${{p}_{k}}^{T}{p}_{k}=1$. The feature vector represents a weight relationship between new variable and the original variable; the new variable $\hat{{\rm{X}}}$ and the original variable X have the same sum of square and sum of variance. The maximum eigenvalue represents the greatest variation after mapping of the original vector. Thus, the weightage produces the most discriminatory data.

13 $$\begin{array}{cc}{\rm{Y}}=\bar{X}+{p}_{k}\hat{X} & ,\,k=1\end{array}$$

Y is the last point situation after PCA processing. After training, points can be connected to obtain a complete contour.

When the conical chamber of drosophila cardiac expanded significantly, the chamber wall was not obvious on the image and noise may appear at the edge. In this case, the level set cost function should be modified by add the energy term produced by the model to effectively limit the contour changes during the iteration.

The level set cost function was modified from eq. (4) as follows:

14 $${\rm{\varepsilon }}(\varnothing )={\rm{\mu }}{R}_{p}(\varnothing )+\lambda {L}_{g}(\varnothing )+\alpha {A}_{g}(\varnothing )+\beta D(\varnothing ,{\varnothing }_{ref})$$

where *ϕ* is the level set function and μ, *λ*, *β* > 0, $\alpha {\epsilon } {\mathcal R}$

Since the Shape Prior-based Level Set Model is to calculate the contour, although the principal component analysis has the characteristics that data has the same total square sum after the processing, the trained model contour size is still different from the actual size. To calculate the difference between the two shapes, the model contour is scaled to the same size as the zero level set, and the energy of the function D in eq. (14) is produced by calculating the distance between the zero level set and the prior model. Calculate the shortest distance from each sample point on the contour to the model contour, and substitute the set of distances into eq. (14). The calculated distance energy function is added to the level set algorithm to correct the deficiency that weak boundary cannot efficiently converge during the iteration.

15 $${\rm{D}}({\rm{X}},{\rm{Y}})=\begin{array}{c}inf\\ x\in X,y\in Y\end{array}d(x,y)$$

In equation (16), *d*(*x*, *y*) represents a Euclidean distance. *inf* is infimum represents the greatest lower bound. To limit the contour, when the model contour is larger than the function contour, the energy is positive energy that makes the function expand outward. On the contrary, the energy is negative, the equation can be rewritten as eq. (16). That is the current contour will close to the nearest point on the model contour.

16 $${\rm{D}}({\rm{X}},{\rm{Y}})={\rm{sign}}(\varnothing ({\rm{y}}))in{f}_{x\in X}d(x,y)$$

**Table:** 

Automated Chamber Segmentation: Shape Prior-based Level Set Model
While *ϕ*_*t*+1_ − *ϕ*_*t*_ > condition of convergence
If current area of zero level set < th
do the original level set
Else
calculate the area of current zero level set
make the model area approximate to the area of zero level set
do a prior model-added level set
End
End

### Drosophila heartbeat counting

The segmentation algorithm proposed by this study can accurately segment the boundary of the heart chamber of Drosophila and apply it to the counting of heartbeats, replacing the traditional dilemma that must be manually counted on B-mode OCT images, and overcoming insufficient amount of information that due to M-mode only consists of one-dimensional images. This study proposes to remove the interference of continuous statistical information of heart chamber area by Gaussian smoothing, taking the local peak as a count. This method also avoids the defect that M-mode’s excessive sensitivity leads to inaccurate calculations, accurately count the heartbeat of Drosophila. The calculation is as shown in the following equation.

17 $${S}_{a}=G({{\rm{A}}}_{m})$$

18 $${\rm{L}}\,[{\rm{k}}]=\{\begin{array}{c}if\,{S^{\prime} }_{a}[k]=0\,\& \,\& \,{S^{\prime\prime} }_{a}[k] < 0=1\\ else=0\end{array},\,k=[2\,\ldots \,M-1]\in N$$

19 $${\rm{heartbeat}}={\rm{sum}}({\rm{L}}\,[{\rm{k}}])$$

where A_*m*_ is continuous statistical information matrix of heart chamber area, G is Gaussian smoothing filter. It is used to suppress small amplitude noise, which avoids the problem of incorrect counting caused by calculating local peaks. M is total number of image.

## Data Availability

All data used for this study are available on request.
